# The dual mechanism of m^6^A demethylase ALKBH5 in regulating energy metabolism during exposure to MC-LR

**DOI:** 10.1038/s41419-025-07791-x

**Published:** 2025-07-03

**Authors:** Xiaoya Sun, Qinmei Tan, Yue Yang, Jia Wei, Xiaodie Zhou, Shanshan Gao, Fei Yang

**Affiliations:** 1 Hunan Province Key Laboratory of Typical Environmental Pollution and Health Hazards, School of Public Health, Hengyang Medical School, University of South China, Hengyang, Hunan Province China; 2 Xiangya School of Public Health, Central South University, Changsha, Hunan China; 3 Department of Radiation Biology, Beijing Key Laboratory for Radiobiology, Beijing Institute of Radiation Medicine, Beijing, China

**Keywords:** Molecular biology, Diseases

## Abstract

Exposure to MC-LR has been shown to cause multiple organ injury, particularly liver injury, and altered energy metabolism is closely linked. As an effective and efficient way to regulate biological gene expression, N(6)-methyladenosine(m^6^A) modification plays an important role in liver injury caused by microcystin-LR(MC-LR) exposure. For the first time, we reveal the dual mechanism by which AlkB homolog 5(ALKBH5) regulates energy metabolism through an m^6^A-YTHDF3-dependent mechanism. After MC-LR exposure, low levels of ALKBH5 increased the m^6^A modification of Phosphoinositide-3-Kinase Regulatory Subunit 1(PIK3R1) and m^6^A methylation was located at A1557. PIK3R1-m^6^A was recognised by YTH N6-Methyladenosine RNA Binding Protein F3(YTHDF3), which reduced the stability of PIK3R1 RNA, thereby inhibiting PIK3R1 expression and ultimately promoting glycolysis. In concert, low-level ALKBH5 inhibit oxidative phosphorylation by down-regulating the expression of Electron Transfer Flavoprotein Dehydrogenase(ETFDH), Electron Transfer Flavoprotein Subunit Alpha(ETFA) and NADH:Ubiquinone Oxidoreductase Complex Assembly Factor 4(NDUFAF4) through an m^6^A-YTHDF3-dependent mechanism. This dual mechanism has been shown to adversely affect cell survival in MC-LR exposed environments by significantly reducing ATP levels. This study reveals for the first time the signalling pathway and molecular mechanism of MC-LR exposure to liver injury through ALKBH5-mediated m^6^A modification, providing new protective and therapeutic principles.

**Subject terms**: m^6^A modification; Oxidative phosphorylation; Glycolysis

**Figure:**
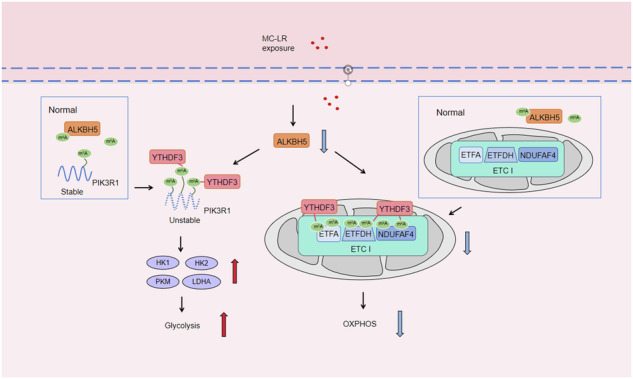
The mechanism of m^6^A demethylase ALKBH5 in regulating energy metabolism during exposure to MC-LR. Created with BioRender.com.

The mechanism of m^6^A demethylase ALKBH5 in regulating energy metabolism during exposure to MC-LR. Created with BioRender.com.

## Introduction

Cyanobacteria blooms from eutrophic waters have become a serious environmental problem worldwide, and cyanotoxins produced by cyanobacteria are a serious threat to ecosystems and public health [1, 2]. Microcystin is a cyclic heptapeptide toxin and more than 200 isomers have been reported, of which MC-LR is the most toxic and widely distributed [3, 4]. In recent years, there has been a surge in reports of MC-LR emerging in estuaries and brackish coastal waters worldwide, including Europe, the Americas, Asia, Oceania and Africa [5]. MC-LR is stable and difficult to degraded, it can enter and accumulate in human body through various ways such as food, water and inhalation, resulting in multi-organ toxicity [6, 7]. Among them, studies have shown that MC-LR is mainly absorbed, transported and accumulated in the liver, resulting in liver damage [8]. MC-LR enters hepatocytes through Organic Anion Transporting Polypeptides(OATPs), causing DNA damage, lipid metabolism disorders, inflammation, cellular autophagy, etc., resulting in liver damage and even tumour formation [9, 10]. Several studies have demonstrated a positive link between exposure to MC-LR and alterations in energy metabolism [11, 12]. Impaired energy metabolism can result in persistent physiological disorders. Under normal circumstances, there is a balance between glycolysis and oxidative phosphorylation(OXPHOS). However, if the cell is damaged, glycolysis is enhanced to produce ATP for enhanced energy supply [13]. In this case, glycolysis enhancement is insufficient to meet energy requirements and can lead to cell death. Altered energy metabolism from mitochondrial oxidative phosphorylation to glycolytic processes affects cell repair, survival, proliferation, metastasis, and regulation of immune homoeostasis, and is an important regulatory mechanism in cellular response to environmental injury [14].

RNA m^6^A modification is one of the most common forms of RNA modification [15], which regulates gene expression by modulating RNA splicing [16], nuclear translocation [17], translational regulation [18], and degradation in a variety of biological processes [19], thus enabling cells to respond rapidly to environmental signals and adapt to the ever-changing microenvironment [20–22]. RNA m^6^A modifications and their biological functions are performed and regulated by m^6^A writers, erasers, and reader proteins. Methyltransferases, include Methyltransferase-like 3(METTL3), Methyltransferase-like 14(METTL14), WT1 Associated Protein(WTAP), RNA Binding Motif Protein 15(RBM15), RNA Binding Motif Protein 15B(RBM15B), Methyltransferase-like 16(METTL16), and others [23–27], catalyse the transfer of methyl groups from s-adenosylmethionine to the N-6 position of adenosine, i.e., the formation of m^6^A modifications [28]. The m^6^A readers, such as the YTH domain-containing proteins, which include YTH domain-containing protein 1(YTHDC1), YTH domain-containing protein 2(YTHDC2), and the family of YTHDF proteins [29, 30]. The YTH structural domains are modules that bind to m^6^A, i.e., it is dependent on m^6^A modification binding to RNA [31]. m^6^A erasers include Fat mass-and obesity-associated protein(FTO) and ALKBH5 [32]. ALKBH5 directly catalyses the RNA adenosine to remove the methyl group, thereby regulating the various RNA functions [33]. ALKBH5 is located in the nuclear speckles, which is the processing site of pre-mRNA, and its depletion leads to global reduction of poly(A) RNAs in this cellular compartment [33]. These three classes of proteins act differently but work together to maintain homoeostasis of m^6^A modification in the cells [34].

m^6^A modifications and related enzymes have been shown to be involved in a number of important processes in response to various stimuli and stresses [35]. Many studies have been carried out on the dynamic changes of m^6^A modifications in the regulation of energy metabolism in the development of various diseases [36–38]. ALKBH5 reduced CKα expression in an m^6^A-dependent manner, which significantly inhibited glucose uptake and lactate production by bladder cancer cells, and reduced intracellular ATP levels, increasing the sensitivity of bladder cancer to chemotherapy [39]. Other studies have shown that ALKBH5 inhibits the glycolytic enzyme PKM2 by mediating the m^6^A modification of circNRIP1 and regulates glycolytic function, thereby affecting lymph node metastasis stage and tumour development in thyroid cancer [40]. The glycolytic enzyme PKM2 was also found to be regulated by ALKBH5, promoting glycolysis and accelerating the development of colorectal cancer [41]. Although the m^6^A modification has been shown to affect the onset and development of the disease by regulating the process of energy metabolism, its effect and mechanism on MC-LR-exposed damaged cells have not been reported.

In this study, we found that after MC-LR exposure, the expression of ALKBH5 in mouse liver tissue and THLE-3 cells was significantly decreased, while its mediated m^6^A modification was significantly increased, which was significantly correlated with MC-LR-induced liver injury. We demonstrated the dual mechanism of ALKBH5 in MC-LR exposure. On the one hand, ALKBH5 downregulation promoted glycolysis by increasing RNA m^6^A modification of PIK3R1 and increasing glycolytic enzymes hexokinase 1(HK1), hexokinase 2(HK2), pyruvate kinase type M(PKM) and lactic dehydrogenase A(LDHA). At the same time, down-regulated ALKBH5 inhibited oxidative phosphorylation by inhibiting ETFDH, ETFA and NDUFAF4, which impair electron transfer chain(ETC) function. During exposure to MC-LR, although ATP levels were elevated by increased glycolysis, but mitochondrial respiration was inhibited, ATP levels were still insufficient to maintain cell growth. For the first time, we reveal the dual mechanism of ALKBH5 and its mediated m^6^A modification in metabolic reprogramming, providing new insights for the development of protection and prevention of MC-LR exposure.

## Results

### MC-LR exposure induces inhibition of hepatocyte growth and increases RNA m^6^A methylation

**Fig. 1:**
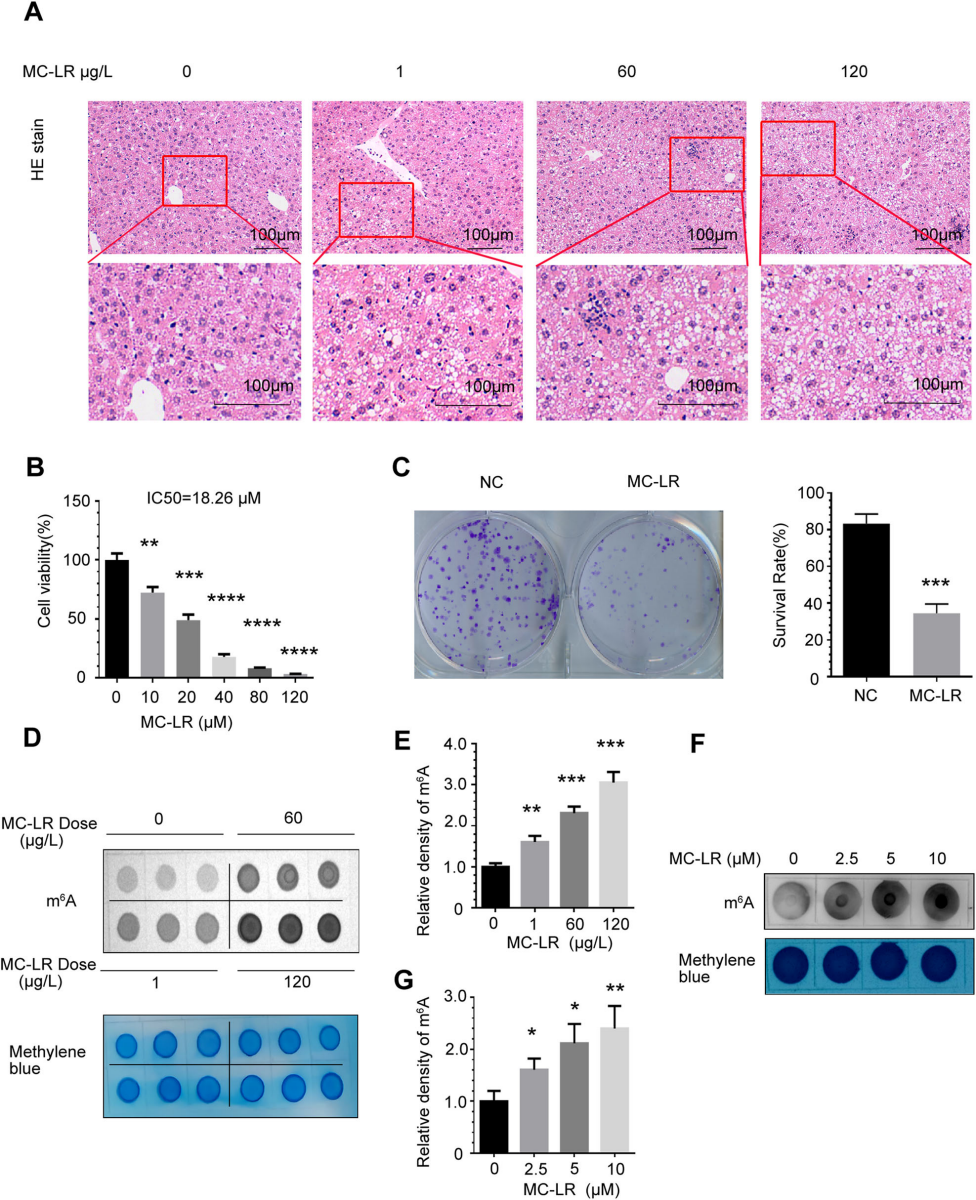
MC-LR exposure induces inhibition of hepatocyte growth and increases RNA m^6^A methylation. **A** Pathological observation of liver tissue from MC-LR exposed mice (HE staining). **B** Effects of MC-LR exposure on proliferation of THLE-3 cells. **C** Effects of MC-LR exposure on colony forming ability in THLE-3 cells. The m^6^A methylation level of the liver tissues of mice with exposure to MC-LR was detected by m^6^A dot blot assay (**D**) and corresponding quantification (**E**). The m^6^A methylation level of total RNA in THLE-3 after 48 h of different doses of MC-LR was detected by m^6^A dot blot assay (**F**) and corresponding quantification(**G**). Data are means ± SD from three independent experiments. *P ≤ 0.05; **P ≤ 0.01; ***P ≤ 0.001; ****P ≤ 0.0001.

MC-LR exposure has been shown to cause multi-organ toxicity, primarily hepatotoxicity [42]. H&E staining of liver sections similarly confirmed this notion, with mice exposed to 1 μg/L, 60 μg/L, and 120 μg/L of MC-LR showing significantly increased liver damage compared to controls(Fig. 1A). In order to confirm the effect of MC-LR exposure on the growth and proliferation of THLE-3 cells, and further confirm the concentration of MC-LR treatment in subsequent experiments. As shown in Fig. 1B, we found that the survival rate of THLE-3 cells significantly decreased with the increase of MC-LR exposure concentration in a dose-dependent effect. Similarly, as shown in Fig. 1C, the colony formation rate of THLE-3 cells was significantly reduced after MC-LR treatment compared to the control. Previous reports have shown that m^6^A modification is a highly effective and efficient way to regulate gene expression, which is undoubtedly a crucial mechanism for damage response and cell survival. To investigate the relationship between m^6^A modification and MC-LR exposure, dot blot assays showed that the levels of m^6^A modification were significantly increased in liver tissue from 1 μg/L, 60 μg/L and 120 μg/L MC-LR exposed mice compared to vehicle control mice(Fig. 1D and E). At the same time, we found that m^6^A modification in the kidney(Supplementary Fig. 1A and B) and duodenum(Supplementary Fig. 1C and D) of MC-LR exposed mice did not change significantly compared with the vehicle control mice. We further confirmed that the m^6^A modification of the THLE-3 cells increased in a dose-dependent manner after treatment with different concentrations of MC-LR (below 1/2 IC50) for 48 hours(Fig. 1F and G).

### ALKBH5 expression is repressed and mediates upregulation of m^6^A modification induced by MC-LR

**Fig. 2:**
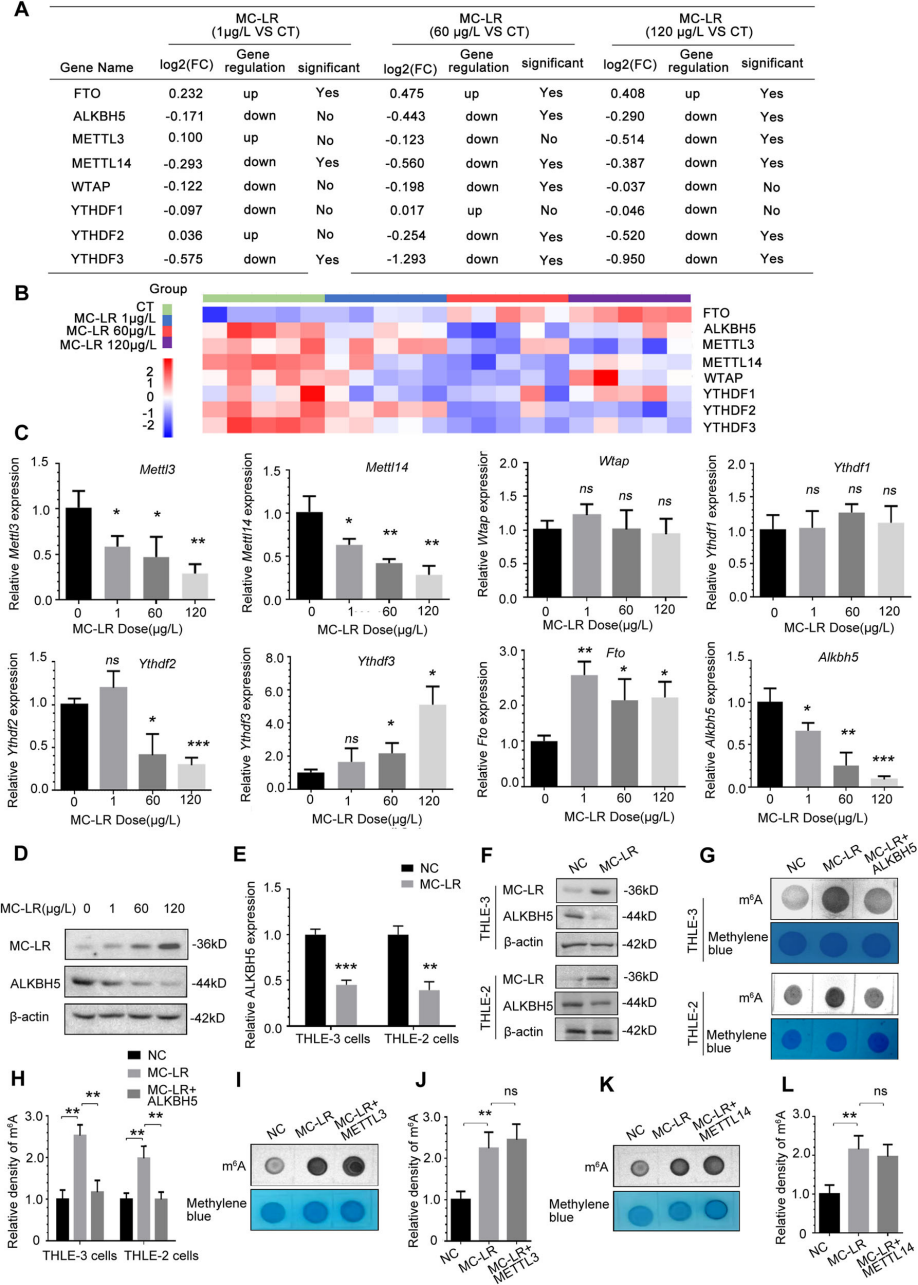
ALKBH5 expression is repressed and mediates upregulation of m^6^A modification induced by MC-LR. **A** In RNA-seq data analysis, m^6^A writers, erasers, and readers in liver tissues from MC-LR exposed mice compared to vehicle control mice. **B** Heatmap of RNA expression of the m^6^A writer, eraser and reader genes. **C** RT-PCR measurements of the enzymes and mediators for RNA m^6^A methylation in liver tissues from MC-LR exposed mice. **D** ALKBH5 protein expression decreased in liver tissue of mice exposed to MC-LR. RT-PCR (**E**) and Western blotting measurements (**F**) of ALKBH5 expression in human liver cells with 5 μM MC-LR exposure. Effect of overexpressing exogenous ALKBH5 vectors on the change in m^6^A level with MC-LR exposure was detected by m^6^A dot blot assay (**G**) and corresponding quantification (**H**). Effect of overexpressing exogenous METTL3 (**I** and **J**) and METTL14 (**K** and **L**) vectors on the change in m^6^A level with MC-LR exposure was detected by m^6^A dot blot assay and corresponding quantification. Data are means ± SD from three independent experiments. *P ≤ 0.05; **P ≤ 0.01; ***P ≤ 0.001.

To reveal the patterns of MC-LR affecting messenger RNA profiles, we performed transcriptome-wide RNA sequencing (RNA-seq) assays of mice liver tissues exposed to MC-LR, including at concentrations of 0, 1, 60 and 120 μg/L, and the analysis of the entire sequencing data can be seen in our previous study [43]. Based on the results of the previous experiments, we first examined m^6^A writer, eraser, and reader proteins in the RNA-seq data. As shown in Fig. 2A, we listed related genes that showed differences in expression after MC-LR exposure, along with heatmaps of these data(Fig. 2B). We further confirmed that after exposure to MC-LR, the expression of ALKBH5 mRNA (Fig. 2C) and protein (Fig. 2D) in mouse liver tissues was most significantly inhibited, and the expression was significantly dose-dependent with the increase in MC-LR concentration. The results combined with Fig. 1D suggest that MC-LR exposure induces upregulation of the m^6^A modification, and only ALKBH5 expression changes were consistent with this trend, but not METTL3, METTL14, YTHDF2 and FTO. Similarly, we found that the RNA and protein expression of ALKBH5 was inhibited after treatment with 5 μM MC-LR in THLE-3 and THLE-2 cells (Fig. 2E and F). To identify the key factors inducing the upregulation of m^6^A modification in MC-LR exposure, we constructed an ALKBH5 overexpression vector and verified its effects on RNA expression(Supplementary Fig. 2A) and m^6^A modification levels (Supplementary Fig. 2B and C). We found that in THLE-3 and THLE-2 cells, dot blot detection showed that MC-LR induced upregulation of m^6^A modification was restored after overexpression of exogenous ALKBH5(Fig. 2G and H). Meanwhile, overexpression of exogenous METTL3(Fig. 2I and J) or METTL14(Fig. 2K and L) had no significant effect on the modification of m^6^A induced by MC-LR. These results suggest that ALKBH5 plays a key role in mediating MC-LR induced upregulation of m^6^A modification.

### ALKBH5 mediates MC-LR exposure-induced altered energy metabolism and cell proliferation inhibition

**Fig. 3:**
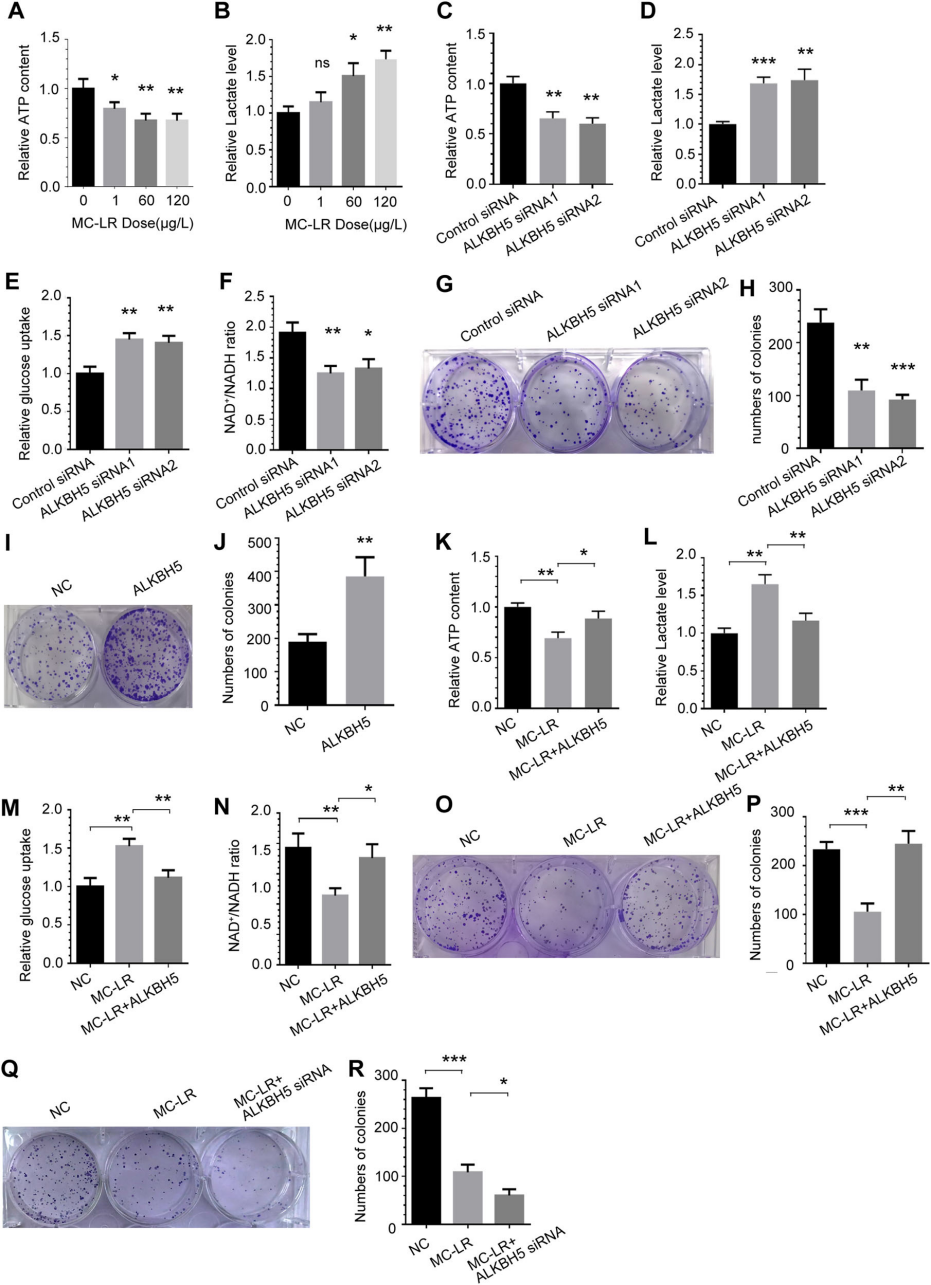
ALKBH5 mediates MC-LR exposure-induced altered energy metabolism and cell proliferation inhibition. Intracellular ATP levels (**A**) and extracellular lactate production (**B**) in liver tissue of mice exposed to MC-LR compared to vehicle control mice. The effect of ALKBH5 knockdown on intracellular ATP levels (**C**), extracellular lactate production (**D**), glucose uptake (**E**) and NAD^+^ /NADH ratios (**F**) was measured compared to control cells. **G**, **H** Effects of knockdown ALKBH5 on the colony-forming abilities of THLE-3 cells. **I**, **J** Effect of overexpression of ALKBH5 on colony forming ability of THLE-3 cells. Effects of ALKBH5 on intracellular ATP levels (**K**), extracellular lactate production (**L**), glucose uptake (**M**) and NAD^+^ /NADH ratios (**N**) of THLE-3 cells following MC-LR exposure. Effect of ALKBH5 overexpression (**O** and **P**) or knockdown (**Q** and **R**) on the colony forming ability of THLE-3 cells after exposure to MC-LR. Data are means ± SD from three independent experiments. *P ≤ 0.05; **P ≤ 0.01; ***P ≤ 0.001.

Previous studies have reported that MC-LR exposure-induced injury responses are associated with glycolysis, although not all mechanistic details are known. To gain further insights, we performed comparative measurements of ATP and lactate secretion in the liver tissue of mice exposed to MC-LR. Exposure to MC-LR resulted in a decrease in ATP levels and an increase in lactate secretion levels in the liver tissues of the mice compared to vehicle control mice (Fig. 3A and B), consistent with a metabolic shift from OXPHOS toward aerobic glycolysis. To investigate the function of ALKBH5, we used specific siRNA molecules to knockdown ALKBH5 in THLE-3 cells (Supplementary Fig. 2D) and found that the m^6^A modification was signifcantly increased(Supplementary Fig. 2E and F). We found that ALKBH5 knockdown significantly decreased ATP levels (Fig. 3C) and the NAD^+^/NADH ratio (Fig. 3F) compared to the control group, whereas extracellular lactate secretion (Fig. 3D) and glucose uptake (Fig. 3E) were significantly increased. Furthermore, knockdown of ALKBH5 signifcantly suppressed the colony-forming ability of THLE-3 cells(Fig. 3G and H), while overexpression of ALKBH5 had the opposite effect (Fig. 3I and J).

To further investigate the effects of ALKBH5 on MC-LR-induced metabolic shift from OXPHOS to aerobic glycolysis, as shown in Fig. 3K, ATP levels were suppressed by MC-LR treatment, whereas overexpression of ALKBH5 greatly attenuated the inhibition of MC-LR on ATP levels. Similarly, overexpression of ALKBH5 significantly reduced the lactate secretion induction of THLE-3 cells caused by MC-LR treatment(Fig. 3L). Glucose uptake assay showed that overexpression of ALKBH5 could significantly inhibit the increase in glucose uptake induced by MC-LR treatment (Fig. 3M). Overexpression of ALKBH5 also increased the NAD^+^/NADH ratio in cells exposed to MC-LR (Fig. 3N). We also found that overexpression of ALKBH5 signifcantly increased the colony-forming ability of THLE-3 cells caused by MC-LR treatment (Fig. 3O and P). In contrast, ALKBH5 knockdown combined with MC-LR treatment further weakened the colony forming ability of THLE-3 cells (Fig. 3Q and R). TThe results suggest that ALKBH5 is a key molecule in MC-LR exposure-induced altered energy metabolism and proliferation inhibition.

### MC-LR exposure inhibits PIK3R1 expression mediated by ALKBH5

**Fig. 4:**
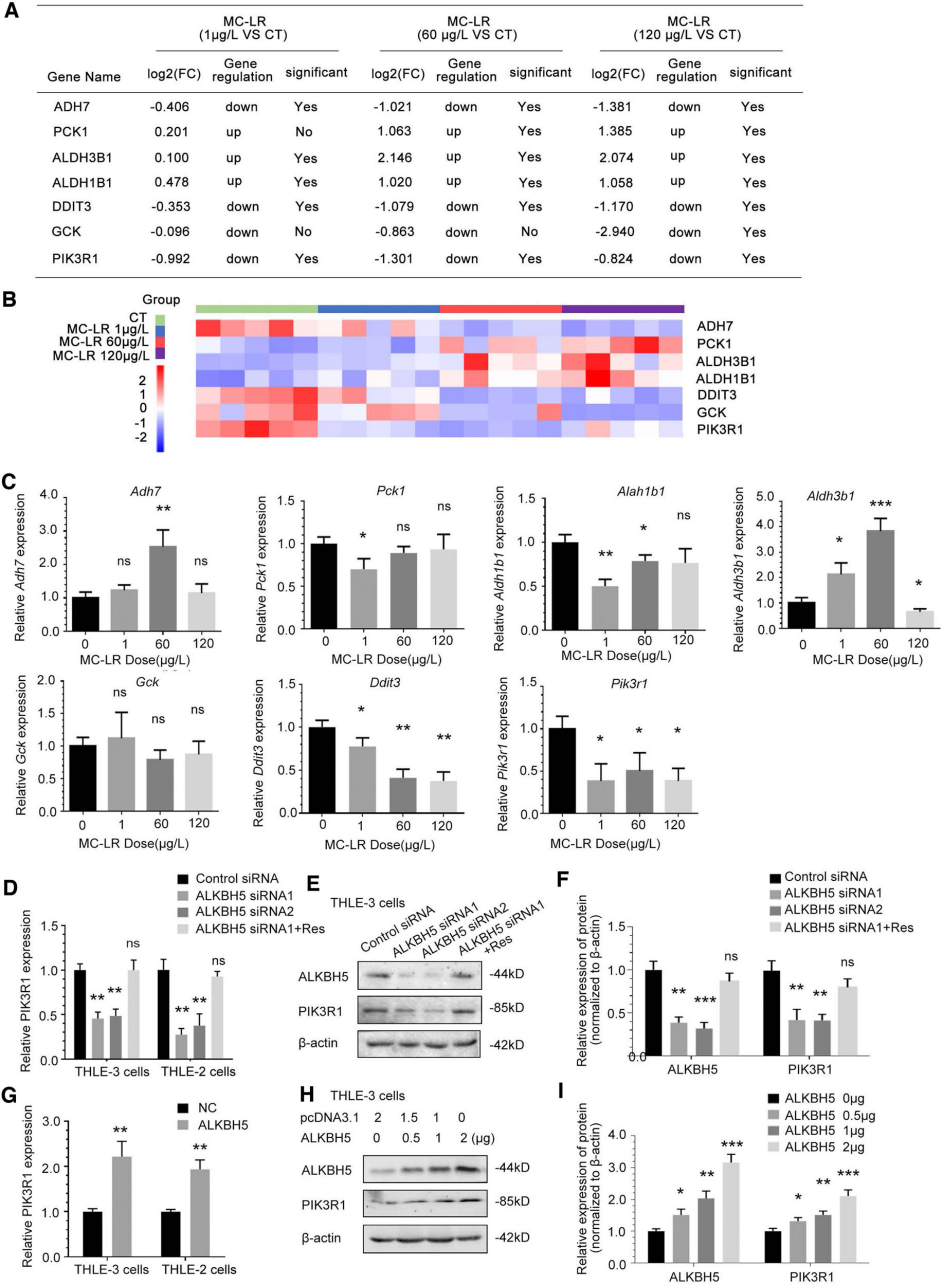

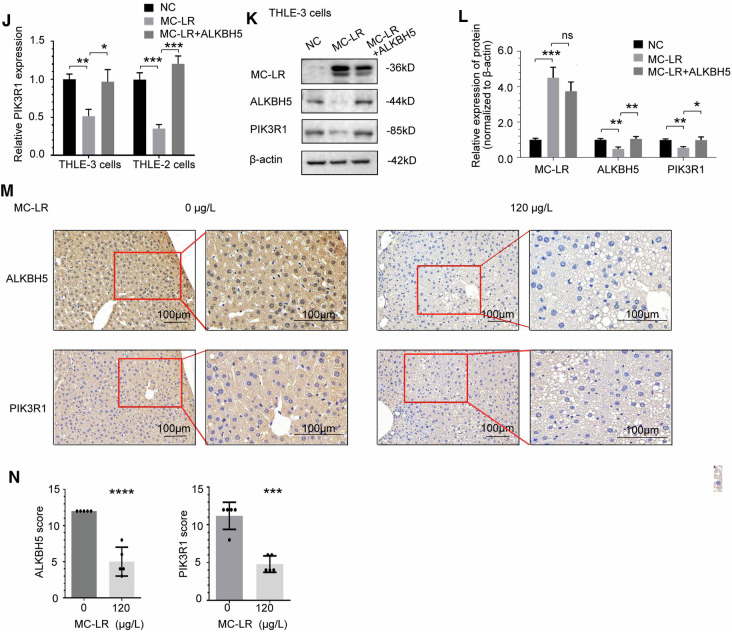
MC-LR exposure inhibits PIK3R1 expression mediated by ALKBH5. **A** Cell proliferation-related genes in liver tissue of mice exposed to MC-LR in RNA-seq data analysis. **B** Heatmap of RNA expression of the cell proliferation-related genes. **C** RT-PCR measurements of the related genes in liver tissue from MC-LR exposed mice. ALKBH5 knockdown reduced PIK3R1 mRNA (**D**) and protein (**E**, **F**) expression. Overexpression of ALKBH5 increased PIK3R1 mRNA (**G**) and protein (**H**, **I**) expression. MC-LR exposure resulted in the downregulation of PIK3R1 mRNA (**J**) and protein (**K**, **L**) expression, which was rescued by overexpression of ALKBH5. **M** Representative images of immunohistochemistry (IHC) analysis of ALKBH5 and PIK3R1 in liver tissue from MC-LR exposed mice. **N** IHC staining scores of ALKBH5 and PIK3R1 in liver tissue of mice exposed to MC-LR. Data are means ± SD from three independent experiments. *P ≤ 0.05; **P ≤ 0.01; ***P ≤ 0.001; ****P ≤ 0.0001.

Based on previous studies, we found differentially expressed genes in the 1 μg/L, 60 μg/L, and 120 μg/L MC-LR groups compared with the control group in the analysis of RNA-seq data, from which genes related to cell proliferation and energy metabolism were selected (Fig. 4A), along with heatmaps of these data(Fig. 4B). Among them, only the RNA expressions of PIK3R1 and DNA Damage Inducible Transcript 3(DDIT3) changed steadily after exposure to different concentrations of MC-LR (Fig. 4C), so we further investigated their correlation with ALKBH5. The expression of PIK3R1 RNA (Fig. 4D) and protein (Fig. 4E and F) was suppressed in human liver cells by ALKBH5 silencing. In contrast, DDIIT3 RNA expression did not change significantly upon ALKBH5 silencing (Supplementary Fig. 2G). Therefore, we chose PIK3R1 as the focus of attention. Consistent with this, PIK3R1 mRNA (Fig. 4G) and protein (Fig. 4H and I) were upregulated in human liver cells overexpressing ALKBH5. The above experiment suggests that PIK3R1 is the responsive target of ALKBH5. Expression of ALKBH5 was repressed in cells after MC-LR exposure, as shown in Fig. 4J-L, we found that PIK3R1 mRNA (Fig. 4J) and protein (Fig. 4K and L) expression was depressed in MC-LR-exposed THLE-3 cellsl, and MC-LR exposure depression of PIK3R1 expression was rescued by overexpression of ALKBH5. To determine the relevance and significance of the ALKBH5-PIK3R1 regulatory axis, immunohistochemical staining was performed in MC-LR-exposed and unexposed mouse liver tissue. The results showed that the expression levels of ALKBH5 and PIK3R1 in the liver of mice exposed to 120 μg/L MC-LR were significantly reduced compared to mice not exposed to MC-LR (Fig. 4M and N).

### ALKBH5 enhances PIK3R1 RNA stability in an m^6^A--YTHDF3-dependent manner and inhibits glycolysis

**Fig. 5:**
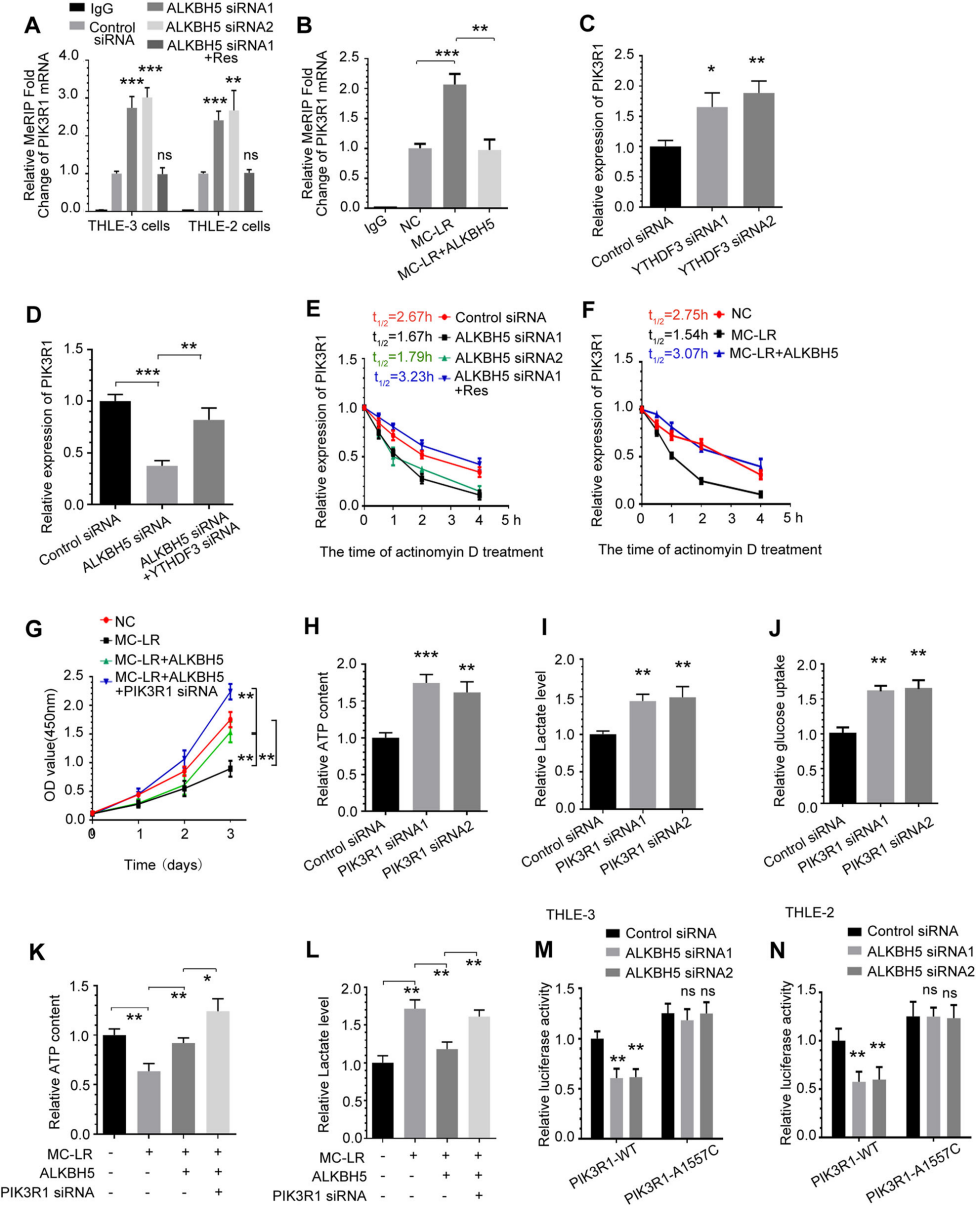
ALKBH5 enhances PIK3R1 RNA stability in an m^6^A-YTHDF3-dependent manner and inhibits glycolysis. **A** PIK3R1 mRNA m^6^A methylation was increased upon knockdown of ALKBH5. **B** PIK3R1 mRNA m^6^A methylation was increased upon MC-LR exposure, which could be rescued by overexpressing ALKBH5. **C** YTHDF3 knockdown increased PIK3R1 mRNA expression. **D** YTHDF3 knockdown can reverse the change in PIK3R1 mRNA expression induced by ALKBH5 knockdown. **E** The half-life of PIK3R1 mRNA was shortened by ALKBH5 knockdown in THLE-3 cells. **F** The half-life of PIK3R1 mRNA was shortened upon MC-LR exposure, which could be rescued by overexpression of ALKBH5. **G** Effects of overexpression of ALKBH5 or simultaneous knockdown of PIK3R1 on cell proliferation inhibition induced by MC-LR exposure. Intracellular ATP levels (**H**), extracellular lactate production (**I**) and glucose uptake (**J**) were measured in PIK3R1 knockdown cells compared to control cells. Effects of ALKBH5 overexpression or PIK3R1 knockdown on intracellular ATP levels (**K**) and extracellular lactate production (**L**) in cells exposed to MC-LR. In THLE-3 (**M**) and THLE-2 (**N**) cells, ALKBH5 knockdown reduced the activity of PIK3R1’s WT. The A1557C mutation of PIK3R1 resulted in increased activity and was not affected by ALKBH5 knockdown. Data are means ± SD from three independent experiments. *P ≤ 0.05; **P ≤ 0.01; ***P ≤ 0.001.

We confirmed that ALKBH5 can positively regulate PIK3R1 expression in previous experiments, so we further explored whether ALKBH5 regulates PIK3R1 in an m^6^A-dependent manner. In order to determine whether PIK3R1 RNA was the direct methylation substrate of ALKBH5, MeRIP-qPCR results showed that m^6^A modification of PIK3R1 was significantly enhanced after knockdown of ALKBH5 in THLE-3 and THLE-2 cells(Fig. 5A). Furthermore, the m^6^A modification of PIK3R1 RNA significantly enhanced after MC-LR exposure, which could be reversed by overexpressing the exogenous ALKBH5 gene (Fig. 5B). To clarify the m^6^A reader of PIK3R1 and determine the regulatory mechanism of m^6^A-dependent ALKBH5, we focused on m^6^A readers with differential expression after MC-LR exposure based on previous experimental results(Fig. 2A and B). Among them, the expression of YTHDF3 increased significantly after MC-LR exposure, which was conducive to identifying the increased PIK3R1 m^6^A modification after MC-LR exposure, so we focused on detecting it. Our results showed that PIK3R1 expression was significantly upregulated after YTHDF3 knockdown(Fig. 5C), which was consistent with our expectations. In addition, knockdown of YTHDF3 reversed the downregulation of PIK3R1 expression induced by ALKBH5 knockdown(Fig. 5D), further demonstrating the important role of YTHDF3 in the PIK3R1-m^6^A-ALKBH5 axis.

As the m^6^A modification plays an important role in the regulation of RNA stability, the half-life of PIK3R1 mRNA was measured to investigate how ALKBH5-mediated m^6^A modification affects PIK3R1 mRNA metabolism. PIK3R1 mRNA degraded faster when ALKBH5 was silenced by siRNA in THLE-3 cells, which could be reversed by overexpressing the exogenous ALKBH5 gene (Fig. 5E). In addition, the half-life of PIK3R1 RNA was shortened after MC-LR exposure, and overexpressing the exogenous ALKBH5 gene could reverse the inhibition of MC-LR on the RNA stability of PIK3R1 (Fig. 5F). These results suggest that ALKBH5-mediated m^6^A modification can regulate the stability of PIK3R1 RNA.

To further determine whether the effect of ALKBH5 in inhibiting cell proliferation induced by MC-LR exposure was related to PIK3R1, we overexpressed ALKBH5 in the presence or absence of PIK3R1 siRNA in THLE-3 cells. We found that overexpression of ALKBH5 weakened the inhibition of cell proliferation induced by MC-LR, while silencing PIK3R1 further enhanced cell proliferation (Fig. 5G). As MC-LR exposure leads to a metabolic shift from OXPHOS to aerobic glycolysis, based on inferences from our observations, we considered whether the ALKBH5/PIK3R1 axis affects glycolytic processes, particularly in the context of MC-LR exposure, but we also considered that ALKBH5 has an additional effect on OXPHOS. As shown in Fig. 5H to J, compared with the control cells, the levels of ATP, extracellular lactate secretion and glucose uptake were significantly increased in PIK3R1 knockdown cells compared to control cells. As we predicted, these results suggest that inhibition of PIK3R1 promotes glycolysis, particularly in the MC-LR exposed environment. Further experiments showed that overexpression of ALKBH5 weakened the inhibition of MC-LR on ATP level in cells, while silencing PIK3R1 further enhanced ATP level on this basis (Fig. 5K). In addition, overexpression of ALKBH5 attenuated the level of MC-LR-induced extracellular lactate secretion, which could be counteracted by silencing PIK3R1(Fig. 5L). In MC-LR exposure, low levels of ALKBH5 promoted glycolysis through inhibition of PIK3R1, which partially contributed to cell energy production, but also suggested that ALKBH5 had an additional role in oxidative phosphorylation.

We then evaluated potential m^6^A modification sites in PIK3R1 and identified the top ten sites with the highest scores using SRAMP (Table S2). All of the ten potential m^6^A sites are located in the 3’ UTR of PIK3R1. To identify which site was modified by the ALKBH5-mediated m^6^A on the PIK3R1, luciferase reporters with wildtype or different mutant PIK3R1 as demonstrated were generated (Supplementary Fig. 3A). Report assays indicate that all other mutant site except A1557C at PIK3R1 were repressed after ALKBH5 silencing (Fig. 5M and N, Supplementary Fig. 3B). These results indicate that A1557 of PIK3R1 is potentially modified by m^6^A. All these results indicate that A1557 of PIK3R1 is the ALKBH5-mediated m^6^A modification site.

### ALKBH5 inhibits glycolytic pathway enzymes through the mediation of PIK3R1

**Fig. 6:**
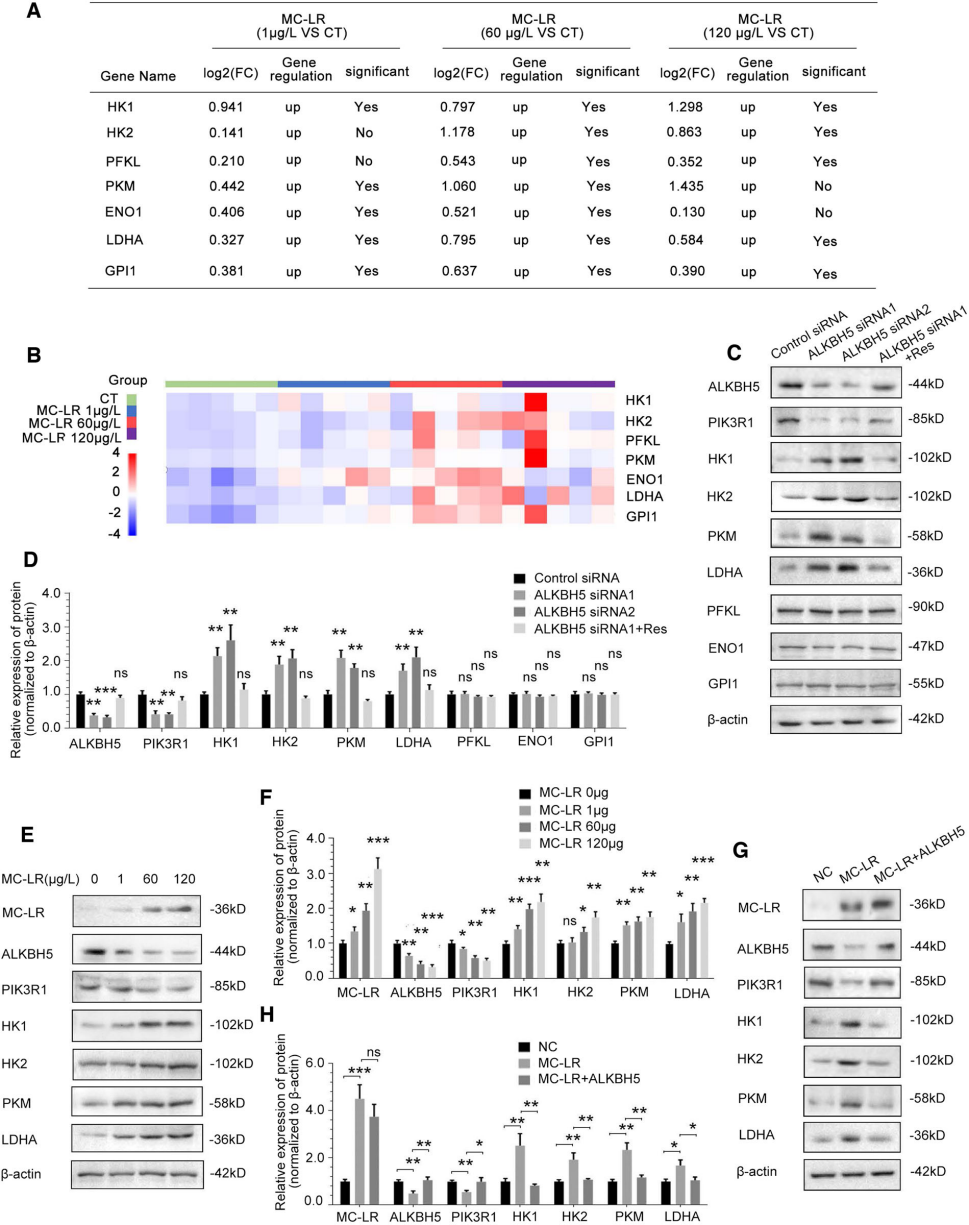

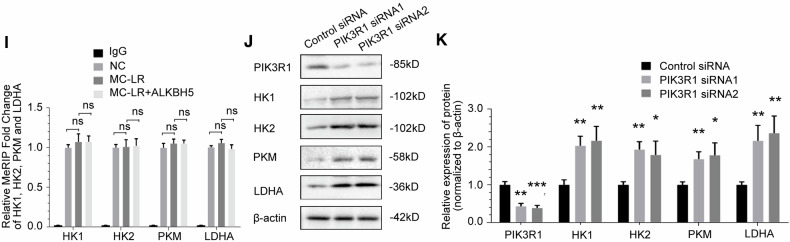
ALKBH5 inhibits glycolytic pathway enzymes through the mediation of PIK3R1. **A** RNA-seq data analysis of glycolytic pathway enzyme genes in liver tissue from MC-LR exposed mice. **B** Heatmap of RNA expression of the glycolytic pathway enzyme genes. **C**, **D** The effect of ALKBH5 knockdown on the expression of PIK3R1 and glycolytic pathway enzymes in THLE-3 cells, as detected by Western blotting and quantified. **E**, **F** The effect of MC-LR exposure on the expression of MC-LR, ALKBH5, PIK3R1 and glycolytic pathway enzymes in mouse liver tissues, as detected by Western blotting and quantified. **G**, **H** The effect of overexpression of ALKBH5 on the expression levels of MC-LR, ALKBH5, PIK3R1 and glycolytic pathway enzymes proteins in THLE-3 cells treated with MC-LR, as detected by Western blotting and quantified. **I** m^6^A modifications of HK1, HK2, PKM and LDHA were not affected by MC-LR exposure and ALKBH5 overexpression. **J**, **K** The effect of PIK3R1 knockdown on the expression of glycolytic pathway enzymes in THLE-3 cells, as detected by Western blotting and quantified. Data are means ± SD from three independent experiments. *P ≤ 0.05; **P ≤ 0.01; ***P ≤ 0.001.

We first considered whether changes in the expression of glycolytic pathway enzymes explained the alterations in glycolytic flux either resulting from MC-LR exposure or ALKBH5 knockdown. Therefore, we chose genes related to glycolytic pathway enzymes as a selection range in RNA-seq assays of MC-LR exposed mouse liver tissues(Fig. 6A), including HK1 [44], HK2 [45], PKM [46], LDHA [47], phosphofructokinase-1, liver type(PFKL) [48], glucose-6-phosphate isomerase(GPI) [49] and enolase 1(ENO1) [50]. Heatmaps of RNA expression of glycolytic pathway enzyme genes are shown in Fig. 6B. Notably, knockdown of ALKBH5 increased the protein levels of PIK3R1, HK1, HK2, PKM and LDHA, but not of PFKL, GPI or ENO1, in THLE-3 cells(Fig. 6C and D), while overexpression of exogenous siRNA-resistant ALKBH5 vectors rescued the upregulated protein levels. We further confirmed that protein expression of HK1, HK2, PKM and LDHA was significantly increased in mice liver tissues compared to control and showed a dose-dependent effect with MC-LR exposure (Fig. 6E and F). MC-LR-induced upregulation of glycolytic pathway enzymes HK1, HK2, PKM and LDHA protein expression was rescued by overexpression of exogenous ALKBH5 (Fig. 6G and H). We further investigated whether ALKBH5 regulates HK1, HK2, PKM and LDHA in an m^6^A-dependent manner. MeRIP-qPCR results showed that m^6^A modifications of HK1, HK2, PKM and LDHA RNA did not change significantly after exposure to MC-LR. In addition, overexpression of exogenous ALKBH5 had no significant effect on m^6^A modification of HK1, HK2, PKM and LDHA(Fig. 6I). The results show that ALKBH5 does not directly regulate HK1, HK2, PKM and LDHA in an m^6^A-dependent manner. In addition, protein expression levels of HK1, HK2, PKM and LDHA were upregulated in THLE-3 cells after knockdown of PIK3R1(Fig. 6J and K). These findings indicated that four crucial glycolytic genes(HK1, HK2, PKM and LDHA) as targets of the ALKBH5/PIK3R1 axis.

### Depresses of ALKBH5 suppresses mitochondrial oxidative phosphorylation during MC-LR exposure

**Fig. 7:**
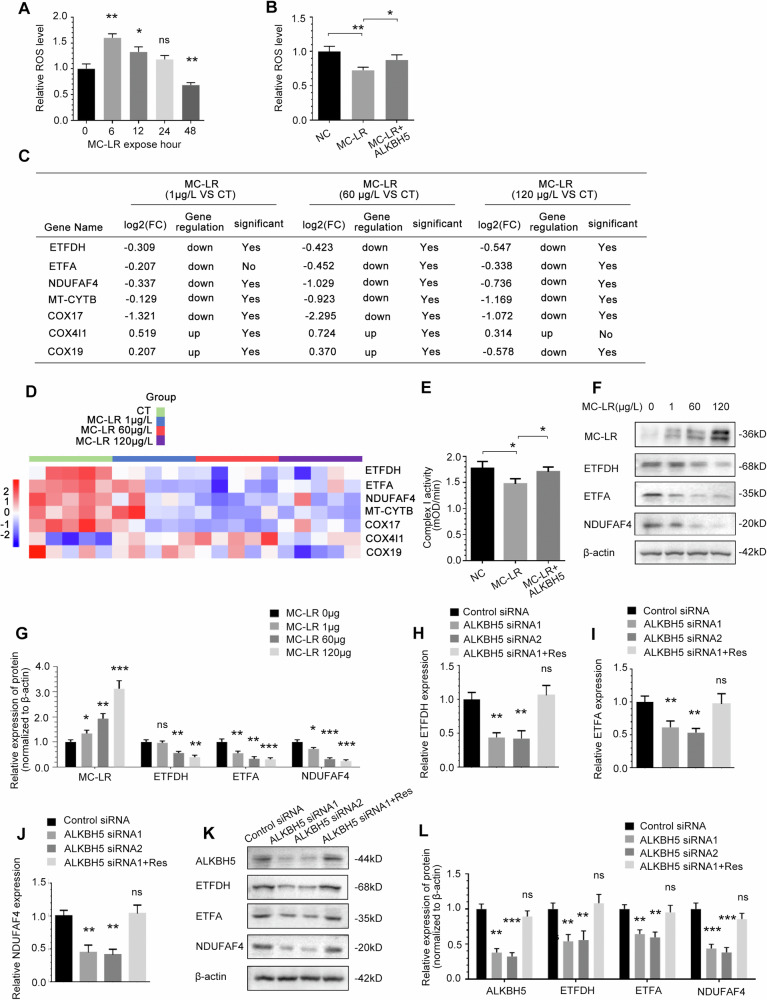
Depresses of ALKBH5 suppresses mitochondrial oxidative phosphorylation during MC-LR exposure. **A** Cellular ROS levels determined in THLE-3 cells cultured under MC-LR exposure for 0, 6, 12, 24, 48 h. **B** Effects of overexpression of ALKBH5 on Cellular ROS levels exposed to MC-LR. **C** Analysis of mitochondrial oxidative phosphorylation enzyme genes in liver tissue of mouse exposed to MC-LR in RNA-seq data analysis. **D** Heatmap of RNA expression of the mitochondrial oxidative phosphorylation enzyme genes. **E** Specific regulation of ETC complex I activity by overexpression of ALKBH5 under MC-LR exposure. **F**, **G** The effect of MC-LR exposure on the expression of ETFDH, ETFA and NDUFAF4 in mouse liver tissue, as detected by Western blotting and quantified. Effect of ALKBH5 knockdown on ETFDH, ETFA and NDUFAF4 RNA (**H–J**) and protein (**K**, **L**) expression in THLE-3 cells. Data are means ± SD from three independent experiments. *P ≤ 0.05; **P ≤ 0.01; ***P ≤ 0.001.

The liver is dependent on mitochondrial oxidative phosphorylation for ATP production, and our experiments consistently showed that MC-LR exposure resulted in a profound decrease in cellular ATP levels. On this basis, we examined the effects of MC-LR exposure on oxidative phosphorylation. Since mitochondrial oxidative phosphorylation as a major source of reactive oxygen species(ROS), we used the detection of cellular ROS levels to indicate mitochondrial respiration. Cellular ROS levels varied in a time-dependent manner during MC-LR treatment, peaking at 6 h, followed by a gradual decrease, and were significantly lower than 0 h levels at 48 h (Fig. 7A). MC-LR treatment inhibited ROS levels in THLE-3 cells, and overexpression of ALKBH5 on this basis significantly increased ROS levels(Fig. 7B). These data indicate that oxidative phosphorylation is inhibited by low levels of ALKBH5 during the long-term MC-LR exposure.

**Fig. 8:**
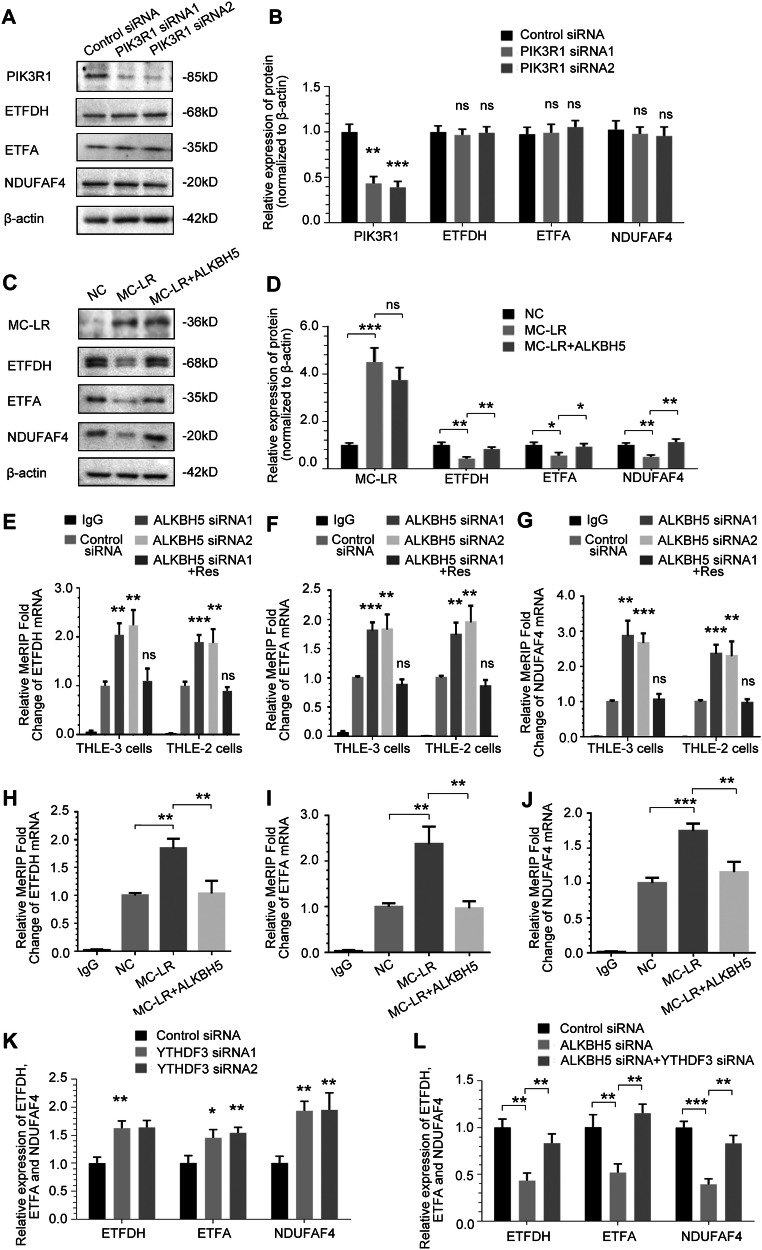
ETFDH, ETFA and NDUFAF4 mRNAs are targets of ALKBH5 demethylation. **A**, **B** The effect of PIK3R1 knockdown on the expression of ETFDH, ETFA and NDUFAF4 in THLE-3 cells, as detected by Western blotting and quantified. **C**, **D** The effect of overexpression of ALKBH5 on the expression levels of ETFDH, ETFA and NDUFAF4 proteins in THLE-3 cells treated with MC-LR,as detected by Western blotting and quantified. **E**–**G** ETFDH, ETFA and NDUFAF4 m^6^A modifications were increased upon knockdown of ALKBH5. **H**–**J** ETFDH, ETFA and NDUFAF4 RNA m^6^A modifications was increased upon MC-LR exposure, which could be rescued by overexpressing ALKBH5. **K** YTHDF3 knockdown increased ETFDH, ETFA and NDUFAF4 mRNA expression. **L** YTHDF3 knockdown can reverse the change in ETFDH, ETFA and NDUFAF4 mRNA expression induced by ALKBH5 knockdown. Data are means ± SD from three independent experiments. *P ≤ 0.05; **P ≤ 0.01; ***P ≤ 0.001.

We analysed the RNA-seq data for enzymes involved in mitochondrial oxidative phosphorylation and selected genes with significant differences, most of which were components of ETC complexes I [51] (Fig. 7C). Figure 7D shows heatmaps of RNA expression of the mitochondrial oxidative phosphorylation enzyme genes. Consistent with our hypothesis, the results show that MC-LR exposure can significantly inhibit the activity of the ETC I complex, and on this basis, overexpression of ALKBH5 can reduce the inhibitory effect of MC-LR(Fig. 7E). We then focused our observations on the components of ETC complexes I. We demonstrated that protein expression of ETFDH, ETFA and NDUFAF4 of the ETC complexes I component was significantly reduced in mice liver tissues compared to control (Fig. 7F and G). we tested the effect of ALKBH5 on the related components of ETC complexes I. QRT-PCR (Fig. 7H, I and J) and western blot analysis (Fig. 7K and L) showed that compared with the control group, ETFDH, ETFA and NDUFAF4 expression was downregulated in THLE-3 cells after siRNA-mediated knockdown of ALKBH5. These alterations were rescued by overexpression of siRNA-resistant ALKBH5. However, the expression of ETFDH, ETFA and NDUFAF4 was not significantly altered after knockdown of PIK3R1, suggesting that they are not regulated by the ALKBH5/PIK3R1 axis(Fig. 8A and B). MC-LR-induced downregulation of ETFDH, ETFA and NDUFAF4 protein expression was rescued by overexpression of exogenous ALKBH5 (Fig. 8C and D).

We further explored whether ALKBH5 regulates ETFDH, ETFA and NDUFAF4 in an m^6^A-dependent manner. The MeRIP-qPCR results showed that m^6^A modification of ETFDH, ETFA and NDUFAF4 mRNA significantly increased in human liver cells after silencing ALKBH5 (Fig. 8E–G). Furthermore, the m^6^A modification of TFDH, ETFA and NDUFAF4 mRNA was significantly increased after MC-LR exposure, which could be reversed by overexpressing the exogenous ALKBH5 (Fig. 8H–J). All these data indicate that ETFDH, ETFA and NDUFAF4 are direct substrates of ALKBH5. We have confirmed that YTHDF3 plays a role in controlling the fate of methylated PIK3R1 mRNA and, based on the mechanism we have identified above, we are continuing to investigate in our studies whether ETFDH, ETFA and NDUFAF4 operate an m^6^A-dependent mechanism via the m^6^A reading protein YTHDF3. Consistent with our expectation, the expressions of ETFDH, ETFA and NDUFAF4 were significantly increased after siRNA inhibition of YTHDF3 (Fig. 8K). In addition, as shown in the Fig. 8L, YTHDF3 knockdown reversed the downregulation of ETFDH, ETFA and NDUFAF4 expression induced by ALKBH5 knockdown. In conclusion, our data suggest that after MC-LR exposure, the increased methylated ETFDH, ETFA and NDUFAF4 can be directly recognised by the m^6^A reader YTHDF3, which is then further dependent on the reduction of the demethylase ALKBH5. The expression of ETFDH, ETFA and NDUFAF4 was reduced by this m^6^A modification mechanism after MC-LR exposure.

## Discussion

Exposure to MC-LR has been shown to cause damage to the liver, stomach [52], intestines [53], brain [54], lungs [55], skin [56], testes [57] and ovaries [58], with liver damage being the most significant effect [42]. Research has shown that the process of energy metabolism during tissue damage is a key determinant of cell function, death or survival [14]. Studies have shown that after long-term exposure to MC-LR, gluconeogenesis-related genes, glycogenolysis-related genes, glycolysis-related genes and glycogenesis-related genes are significantly altered, which impairs liver energy metabolism and causes persistent physiological disorders [59]. Up to now, the regulatory mechanisms of energy metabolism in environmental stresses, especially in MC-LR exposure, have not been well described. As with other studies, this study found that exposure to MC-LR significantly increased liver tissue damage in mice and inhibited cell proliferation. Further studies showed that after MC-LR treatment, ATP levels and the NAD^+^/NADH ratio decreased, while lactate secretion levels and glucose uptake increased. To our surprise, ROS levels increased within a short period of time (within 24 hours) after MC-LR treatment and decreased significantly after 48 hours, which together indicated a metabolic shift from OXPHOS to aerobic glycolysis. This change in energy metabolism is related to the cell’s response to stimulating environmental conditions. Although glycolysis is partially promoted, the significant inhibition of OXPHOS eventually leads to a shortage of ATP, resulting in increased cell damage.

m^6^A modification is a dynamic process that causes cells to respond quickly to environmental signals. In this study, the effect of m^6^A modification on MC-LR exposure was investigated for the first time, and it was found that RNA m^6^A modification was significantly increased after MC-LR exposure, and ALKBH5, as a key m^6^A erasers, played a crucial role in it. Many studies have indicated that ALKBH5 plays an important role in energy metabolism the glycolysis process and disease development through m^6^A modification that interferes with CK2α [39], PKM2 [41], GLUT4 [60] and ENO1 [61] signalling. We published two mechanisms for ALKBH5 for the first time. ALKBH5 was inhibited after MC-LR exposure and then promoted glycolysis by inhibiting PIK3R1. On the other hand, low levels of ALKBH5 inhibited OXPHOS. The overall effect of ALKBH5 was shown to favour hepatocyte growth, and high levels of ALKBH5 can improve cell survival in MC-LR-exposed environments.

Aerobic glycolysis plays a crucial role in providing rapid energy in the form of ATP and producing biosynthetic intermediates, so cells accelerate glycolysis to achieve new functions in response to various environmental stimuli [62]. ALKBH5 does not directly target glycolytic enzymes, but maintains RNA stability by removing the PIK3R1 RNA m^6^A modification and indirectly regulates the glycolytic enzymes HK1, HK2, PKM and LDHA via PIK3R1. PIK3R1 is the regulatory subunit of phosphoinositide 3-kinases (PI3K), encodes the predominant regulatory subunit P85α of class I PI3K, and inhibits the catalytic activity of P110α kinase [63]. As a regulatory subunit of PI3K, PIK3R1 plays an important role in the regulation of metabolic homoeostasis [64, 65]. We identified HK1, HK2, PKM and LDHA as direct downstream targets of this ALKBH5/PIK3R1 pathway, which affects the efficiency of the glycolytic enzyme cascade. Thus, activation of glycolysis by inhibiting ALKBH5 during MC-LR exposure appears to be a means of maintaining ATP levels to support cell survival. However, it was clearly opposite to the overall inhibitory effect of low ALKBH5 levels on cell proliferation during MC-LR exposure. We therefore investigated other functions and functional mechanisms of ALKBH5. Other studies have found that an increase in aerobic glycolysis under stimulating conditions is often accompanied by a decrease in OXPHOS [66]. The initial response to MC-LR exposure was an increase in cellular ROS levels at 6 h, followed by a gradual decline after 48 h. At the same time, we found that overexpression of ALKBH5 could significantly increase ROS levels in cells treated with MC-LR exposure. The main sites of ROS generation are the ETC respiratory complexes I, II and III [67]. We analysed RNA-seq data for enzymes involved in mitochondrial oxidative phosphorylation and found that most of the genes with significant differences after MC-LR exposure were components of the ETC complex I. Our results also showed that the activity of the ETC I complex decreased significantly after MC-LR exposure. ALKBH5 can clearly regulate the protein levels of ETFDH, ETFA and NDUFAF4 of complex I. It is pertinent to note that ALKBH5 does not regulate the complex I component through PIK3R1. However, further details of other mitochondrial ETC perturbations mediated by ALKBH5 under MC-LR exposure conditions need to be investigated. After MC-LR exposure, the expression of ETFDH, ETFA and NDUFAF4 was significantly decreased, while the modification of RNA m^6^A was significantly increased, which could be reversed by overexpression of ALKBH5. ALKBH5 inhibits OXPHOS by targeting ETFDH, ETFA, and NDUFAF4 signals.

m^6^A readers have been reported to be involved in the control of mRNA fate, and YTHDF3 has been shown to promote the progression of many tumour types, but little is known about its effects on cells under environmental cues. Our data show for the first time that YTHDF3 regulates PIK3R1, ETFDH, ETFA and NDUFAF4 in an m^6^A-dependent manner to promote glycolysis and reduce OXPHOS to achieve cellular inhibition upon MC-LR exposure. These results partially explain the deleterious effects of MC-LR exposure on cells. However, further molecular mechanisms of m^6^A methylation and energy metabolism warrant extensive investigation.

In conclusion, this study, for the first tim,e identified and clarified the important regulatory role of ALKBH5 in MC-LR exposure by mediating RNA m^6^A modification. ALKBH5 regulates energy metabolism through the (PIK3R1, ETFDH, ETFA and NDUFAF4)-m^6^A-YTHDF3 axis, revealing its adverse effects on cells under the influence of the environment. This new aspect of ALKBH5 regulation of energy metabolism function reveals the importance of m^6^A modification and energy metabolism regulation in mediating MC-LR-induced liver injury, providing favourable evidence and insights for the development and precise application of MC-LR protectants.

## Materials and methods

### Cell culture

The cell lines used in this study (Human THLE-3 and THLE-2 cell line) were obtained from Cancer Research Institute of Central South University (Changsha, China), maintained in our laboratory and cultured at 37 °C under 5% CO_2_ in a humidified incubator. THLE-3 and THLE-2 cells were grown in DMEM(HyClone) containing 10% FBS (Gbico) and 1% penicillin‒streptomycin. Unless otherwise noted, the experimental cells were THLE-3 cells and cells for MC-LR experimental treatments were treated with 5μm MC-LR for 48 hours.

### Animals and treatments

The animal experiments were approved, XYGW-2018-41, by the Animal Care and Use Committee of the Central South University, and proceeded by the Laboratory Animal Guideline of Welfare and Ethics of China. Forty C57BL/6 mice were randomly divided into four groups and fed MC-LR drinking water at 0, 1, 60 and 120 μg/L for 12 months, in accordance with our previously published study [68]. Ten mice were included in each experimental group. After 12 months, liver tissue samples were collected after the mice were killed for subsequent testing.

### Plasmid, siRNA

The coding sequences (CDS) of ALKBH5 were cloned into pcDNA3.1 to generate an overexpression plasmid. The CDS of METTL3 was cloned into pcDNA3.1. The CDS of METTL14 was cloned into pEGFPC1. The 3’UTR of PIK3R1-WT was cloned into pmirGLO to generate a fusion reporter genes plasmid. The PIK3R1 3’UTR mutant plasmid was obtained by PCR amplification using pmirGLO-PIK3R1-WT as a template and joined to the vector.

For ALKBH5 knockdown, two synthesized duplex RNAi oligos targeting human mRNA sequences from Sigma were used. (si-ALKBH5-1:5′-UCAGAUCGCCUGUCAGGAATT-3′, si-ALKBH5-2: 5′-GGAUAUGCUGCUGAUGAAATT-3′). For PIK3R1 knockdown,two synthesized duplex RNAi oligos targeting human mRNA sequences from Sigma were used (si-PIK3R1-1:5′-GCAGCCGUUUACAGUGAAATT-3′,si-PIK3R1-2:5′-GCUGGUUAAAUGGCUAUAATT-3′). For PIK3R1 knockdown, two synthesized duplex RNAi oligos targeting human mRNA sequences from Sigma were used (si-YTHDF3-1:5′-GGA CGU GUG UUU AUA AUU ATT-3′, si-YTHDF3-2:5′- GCAGUGGUAUGACUAGCAUTT-3′). A scrambled duplex RNA oligo (5′-UUCUCCGAACGUGUCACGU) was used as RNA control.

Transfected using Lipofectamine 2000 reagent with siRNA negative control (siNC), siRNAs, vector control, or plasmid construct. The working concentration of siRNA was 50 nM and the incubation time is 24 hours. Transfection was performed using Lipofectamine 2000 reagent (Invitrogen) with vector control, plasmid construct, siRNA negative control (siNC), or siRNAs according to the manufacturer’s instructions.

### RNA extraction and quantitative RT-PCR analysis

Total RNA was isolated using TRIzol (Life Technologies, Shanghai, China). RNA was reverse-transcribed into cDNA with Superscript III reverse transcriptase (Vayzme, Nanjing, China) according to the manufacturer’s instructions. Quantitative real-time PCR analysis was performed with 1 μL of cDNA using ChamQ Universal SYBR qPCR Master Mix (Vazyme, Nanjing, China). The quantitative PCR primers were listed in Table S1.

### m^6^A dot blot assay

RNA (450 ng) was cross-linked onto a nylon membrane using UV, then stained with 0.02% methylene blue for 5 min and washed with PBST for 15 min until the background colour became thinner. The membrane was then blocked with 5% nonfat dry milk (1x PBST) for 1 hour and incubated overnight at 4 °C with specific anti-m^6^A antibody(Proteintech,68055-1). HRP-conjugated goat anti-rabbit IgG was then added to the membrane and incubated for 1 hour at room temperature, washed with PBST for 15 minutes and and the blot was visualized by using Luminata Forte Western HRP substrate (Millipore, Darmstadt, Germany).

### m^6^A methylated RNA immunoprecipitation PCR(MeRIP-qPCR)

The RiboMeRIP m^6^A Transcriptome Profiling Kit (RIBO, C11051-1) was used to measure the m^6^A content in the RNAs. The chemically fragmented RNAs were incubated with m^6^A-specific antibodies for immunoprecipitation and then analysed by qPCR for enrichment of m^6^A-containing RNAs, which was used to calculate RNA m^6^A modifications. The MeRIP-qPCR primers were listed in Table S1.

### Hematoxylin-eosin Staining

After the mice were killed, liver tissues were collected, washed with saline, dried and weighed. Liver samples were fixed overnight at 4 °C with 4% paraformaldehyde, embedded in paraffin, and 4 µm sections were stained with HE.

### Immunohistochemistry analysis

After deparaffinization and antigen retrieval, the array was incubated with anti-ALKBH5 antibody (dilution 1:200, Proteintech, 16837-1-AP) or anti-PIK3R1 antibody (dilution 1:200, Proteintech, 60225-1-Ig) at 4 °C overnight. The section was rinsed in PBS and incubated with HRP-labelled goat anti-rabbit or anti-mouse IgG for 30 min at room temperature. The outcome signals were scored according to the percentage of positive cells and staining intensity. Staining intensity was assessed on a scale from 0–3 (0 = negative, 1 = weak, 2 = moderate, 3 = strong), and the percentage of positive cells (0, <5%; 1, 6–25%; 2, 26–50%; 3, 51–75%; and 4, 76–100%) was assessed semiquantitatively. The final scores (0–12) were then calculated by multiplying these 2 values.

### Proliferation assay

Cells (1.5 × 10^3^) were seeded into 96-well plates and cell proliferation was detected by the addition of 10 μL/well Cell Counting Kit-8(CCK8) solution after incubation for various times, followed by incubation at 37 °C for 3 h. Absorbance at 450 nm was recorded by a microplate reader.

### RNA stability assay

To assess RNA stability, the cells were incubated with actinomycin D(Santa Cruz Biotechnology) at 5 mg/mL for the indicated times. The cells were collected, and RNA samples were extracted for qPCR analysis.

### Western blotting analysis

Protein was extracted with RIPA buffer containing 5 mM EDTA, PMSF and phosphatase inhibitor cocktail. About 40 μg total proteins were resolved by SDS-polyacrylamide gel electrophoresis and transferred onto polyvinylidene fluoride (PVDF) membrane (Merck Millipore Ltd., Burlington, MA, USA), blocked with 5% non-fat milk at room temperature for 2 h, and incubated with primary antibodies overnight at 4 °C. After being washed three times, the membrane was incubated with goat anti-mouse IgG (H + L) HRP conjugate or goat anti-rabbit IgG (H + L) HRP conjugated secondary antibodies for 1 h at room temperature, and the blot was visualized by using Millipore’s Luminata Forte Western HRP substrate.

The detection antibodies were as follows: ALKBH5 antibody (Proteintech, 16837-1-AP); PIK3R1 antibody (Proteintech, 30092-1-AP); MC-LR antibody(Alexis Corporation); β-actin antibody(Cell Signalling Technology, 4967); HK1 antibody (Proteintech, 15656-1-AP); HK2 antibody (Proteintech, 22029-1-AP); PKM antibody (Proteintech, 25659-1-AP); LDHA antibody (Proteintech, 21799-1-AP); ETFDH antibody (Proteintech, 11109-1-AP);NDUFAF4 antibody (Proteintech, 26003-1-AP); Microcystin-LR monoclonal antibody (Enzo Life Sciences, ALX-804-320-C200). The Images of all blots were listed in Supplemental Material.

### Dual-luciferase reporter assay

Luciferase reporter assay was performed with the Dual-Luciferase Reporter Assay System (Promega, E1910) according to the manufacturer’s descriptions. Cells were seeded into 24-well plates 1 day before the transfection.

To evaluate the potential roles of m^6^A site in PIK3R1 expression, the wild type or mut type of PIK3R1 was inserted behind the F-Luc coding region. Both the pmirGLO-PIK3R1-WT and pmirGLO-PIK3R1-Mut were transfected into wild-type or ALKBH5 knockdown cells for 24 h. Renilla Luciferase (R-Luc) was used to normalize firefly luciferase (F-Luc) activity. Each group was performed at least three times.

### Metabolite assays

Cellular ATP, extracellular lactate levels, Cellular glucose uptake, NAD^+^/NADH ratio, ETC Complex I OXPHOS Activity, and ROS were measured using the ATP Content Assay Kit(Sangon Biotech, D799646), Lactic Acid (L-LA) Content Assay Kit(Sangon Biotech, D799099), Glucose Uptake Assay Kit(Abcam, ab136956), NAD^+^/NADH Assay Kit(Abcam, ab176723), MitoTox Complex I OXPHOS Activity Microplate Assay (AbCam, ab109903), and ROS Assay Kit (Nanjing jiancheng bioengineering institute, E004-1-1), respectively, according to the manufacturer’s instructions.

### Transcriptome sequencing and data analysis

Sequencing libraries were generated using the Illumina NEBNext® UltraTM RNA Library Prep Kit (NEB, USA) and index codes were added to attribute sequences to each sample. Paired-end sequencing of the library was performed on a HiSeq 4000 platform (Illumina, USA). Quality evaluation of the raw reads was conducted with FastQC_v.0.11.3. Adapters and low-quality reads of raw data were cleaned via Trimmomatic software (version 0.32) based on the sequence quality score. Then, the high-quality reads were employed for the transcriptome assembly using the Trinity software with default parameters.

### Quantification and statistical analysis

Statistical analyses were performed in GraphPad Prism 6, and data are presented as the mean ± SEMs or SDs. Unpaired two-tailed Student’s t-test was used to to compare differences between two groups. One-way analysis of variance with multiple comparison tests was used to compare three or more groups.

## Supplementary information

Supplement table and figur

Images of all blots

## Data Availability

All data needed to evaluate the conclusions in the paper are presented in the paper and/or in the Supplementary Materials. Additional data related to this paper may be requested from the authors.
