# Supplementary material for: The dual mechanism of m6A demethylase ALKBH5 in regulating energy metabolism during exposure to MC-LR
Source: Cell Death Dis. 2025 Jul 3;16(1):489. doi: 10.1038/s41419-025-07791-x (PMC12229691; doi:10.1038/s41419-025-07791-x)
Supplement: Supplementary file 1 — Supplement table and figur [file 41419_2025_7791_MOESM1_ESM.docx]

**Supplemental table lists of primers, related sequences**

**S1. Primers used for qRT-PCR and MeRIP-PCR**

| **Primer Name** | **Primer sequence (5’—3’)** | |
| --- | --- | --- |
|  | **Forward** | **Reverse** |
| METTL3(qRT-PCR) | TTGTCTCCAACCTTCCGTAGT | CCAGATCAGAGAGGTGGTGTAG |
| METTL14(qRT-PCR) | GAGTGTGTTTACGAAAATGGGGT | CCGTCTGTGCTACGCTTCA |
| WTAP(qRT-PCR) | CTTCCCAAGAAGGTTCGATTGA | TCAGACTCTCTTAGGCCAGTTAC |
| YTHDF1(qRT-PCR) | ATACCTCACCACCTACGGACA | GTGCTGATAGATGTTGTTCCCC |
| YTHDF2(qRT-PCR) | ACTTTGAGCCCTACCTTACTGGA | CAATGGACGGCGGGTAATA |
| YTHDF3(qRT-PCR) | TGACAACAAACCGGTTACCA | TGTTTCTATTTCTCTCCCTACGC |
| FTO(qRT-PCR) | TCAGCAGTGGCAGCTGAAAT | CTTGGATCCTC ACCACGTCC |
| ALKBH5(qRT-PCR) | AGTTCCAGTTC AAGCCCATC | GGCGTTCCTTAATGTCCTGAG |
| ALDH1B1(qRT-PCR) | AGCCTCTGTTCAAGTTCAAG | CCTTAAAGCCTCCGAATGG |
| DDIT3(qRT-PCR) | GCGACAGAGCCAGAATAACAGC | TTCTGCTTTCAGGTGTGGTGGT |
| ADH7(qRT-PCR) | ATGGGCACCGCTGGAAAAG | TAACACGGACTTCCTTAGCCT |
| PCK1(qRT-PCR) | CATTGCCTGGATGAAGTTTGACG | GGGTTGGTCTTCACTGAAGTCC |
| ALDH3B1(qRT-PCR) | GAACTACCCCGTGAACCTGAC | ACCTTCTCCGTGCCCTTACTA |
| PKM2(qRT-PCR) | TCACCAAGTCTGGCAGGTCTG | CATTCATGGCAAAGTTCACCCGGA |
| PFKL(qRT-PCR) | GCTACCGTGGACCTGGAG | CCACTCGTTGCGGAAGAT |
| β-actin(Human) | CAGCCTTCCTTCCTGGGCAT | ATTGTGCTGGGTGCCAGGGCAG |
| β-actin (Mice) | GATGGCCACTGCCGCATCCTC | GGTCTTTACGGATGTCAACGTCAC |
| LDHA(qRT-PCR、MeRIP) | GCTCCCCAGAACAAGATTACAG | TCGCCCTTGAGTTTGTCTTC |
| ETFDH(qRT-PCR、MeRIP) | ACACTGTTGGTTGGCCCTTG | GTCCTCATATTCAGTTACATGGAGT |
| ETFA(qRT-PCR、MeRIP) | AGCTGAGCATGCAAATGATTCC | TCACAAATGTGTCAGGTGACTT |
| NDUFAF4(qRT-PCR、MeRIP) | GACACCCCTCTACCAACAGC | ACCTGCAAGGAAGACACAGG |
| NDUFAF4(qRT-PCR) | AGGAGCACCGGAGTCAGTAT | TTGCCTTTGGGAATGTCCGT |
| PIK3R1(qRT-PCR、MeRIP) | AAGAAGTTGAACGAGTGGTTGG | GCCCTGTTTACTGCTCTCCC |
| PKM(qRT-PCR、MeRIP) | CTCGGGCTGAAGGCAGT | AATTGCAAGTGGTAGATGGCA |
| HK1(qRT-PCR、MeRIP) | ATGGCCTCTCCCGGGATTAT | GGGGTGTCGTAGACCTCAGA |
| HK2(qRT-PCR、MeRIP) | GCCTTCGGGGACAATGGATGC | TCTGCTTGCCGGGGTTGAGTG |

**S2. Analysis of potential m^6^A modification site of PIK3R1**

| **Position** | **Sequence context** | **Score**  **(binary)** | **Score**  **(spectrum)** | **Score**  **(combined)** |
| --- | --- | --- | --- | --- |
| 1557 | AAGCCAUCUUUCUUUGGAUGGGACUAGAGCUUUCUUUCACAAAAA | 0.88 | 0.828 0.850 | 0.850 |
| 1742 | GCAAAAAUCUCUGCGUGCAGGGACAAAGAGGCCUUUAACCAUGGU | 0.865 | 0.805 | 0.819 |
| 1807 | GCUUUACCAGCUGAAAGUUGGGACUCUGGAGAGCGGAGGAGAGAG | 0.895 | 0.804 | 0.846 |
| 1901 | UGGAUGUUGCUGUGCACGGUGGACCCAGACACAUCGCACUGUGGA | 0.813 | 0.855 | 0.810 |
| 1939 | CUGUGGAUUAUUUCAUUUUGUAACAAAUGAACGAUAUGUAGCAGA | 0.740 | 0.841 | 0.753 |
| 2104 | ACAGAAAAAUGGAGUUUGGAAAACAGGACUUAAAAUGACAUUCAG | 0.726 | 0.852 | 0.756 |
| 2109 | AAAAUGGAGUUUGGAAAACAGGACUUAAAAUGACAUUCAGUAUAU | 0.917 0.917 | 0.862 | 0.892 |
| 3172 | AGUCAUACUGUCACUGCUCUGGACUGUGUGGAGCUCGCUAAAGUC | 0.756 | 0.567 | 0.667 |
| 3660 | CAAAAGUCAGUCUUAUAGCAAGACUGUUAGCCCUCAAACUUGACU | 0.685 | 0.519 | 0.617 |
| 4475 | GUAUAUAUGUAUAUGUGCAUGGACUGUGUUUCCAGUACACCUUUC | 0.724 | 0.401 | 0.596 |


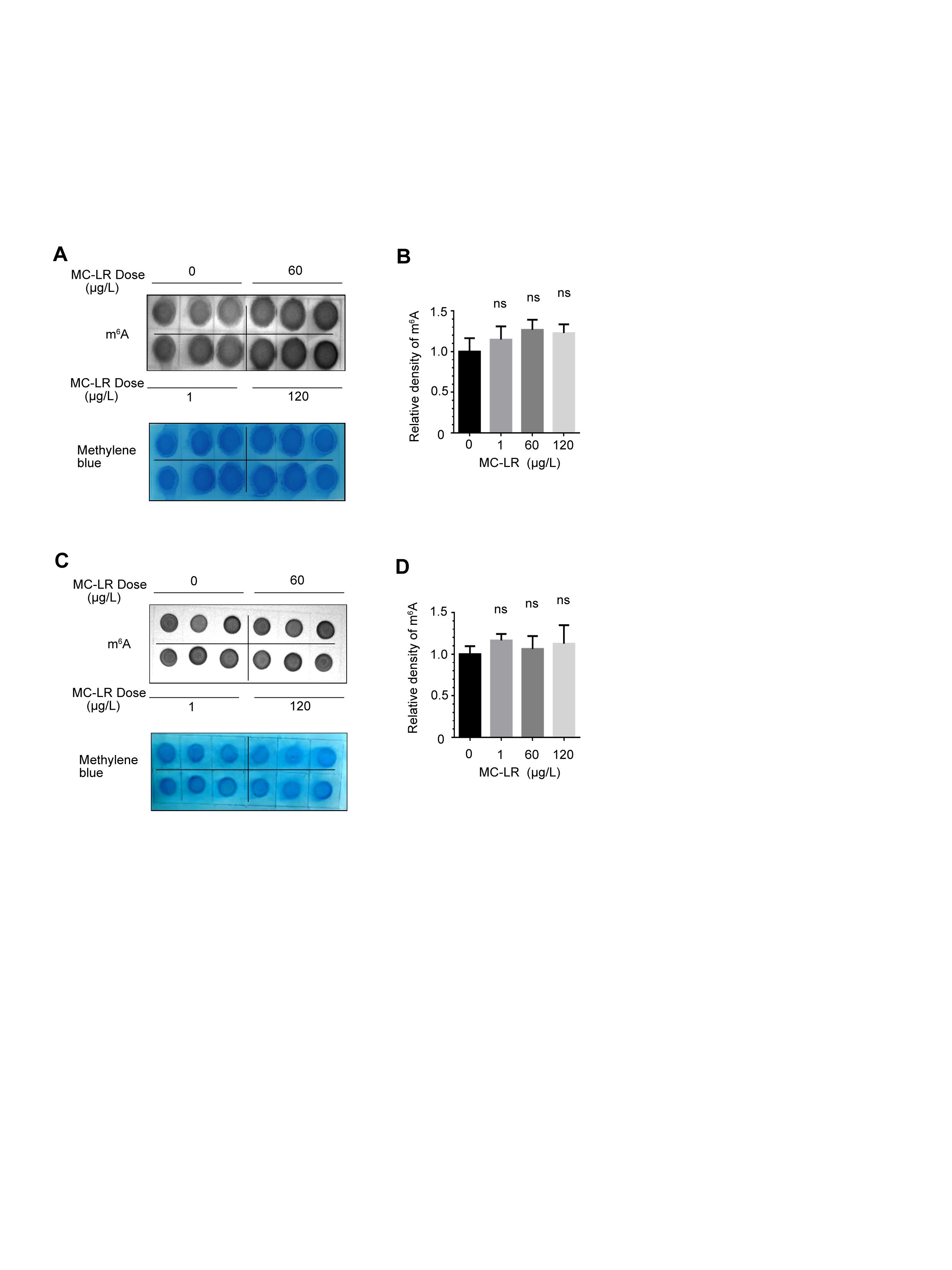


Supplementary Figure 1. **A and B**, The m^6^A methylation level of the kidney tissues of mice with exposure to MC-LR was detected by m^6^A dot blot assay(**A**) and corresponding quantification(**B**). **C and D**, The m^6^A methylation level of the duodenum tissues of mice with exposure to MC-LR was detected by m^6^A dot blot assay(**C**) and corresponding quantification(**D**). Data are means ± SD from three independent experiments.


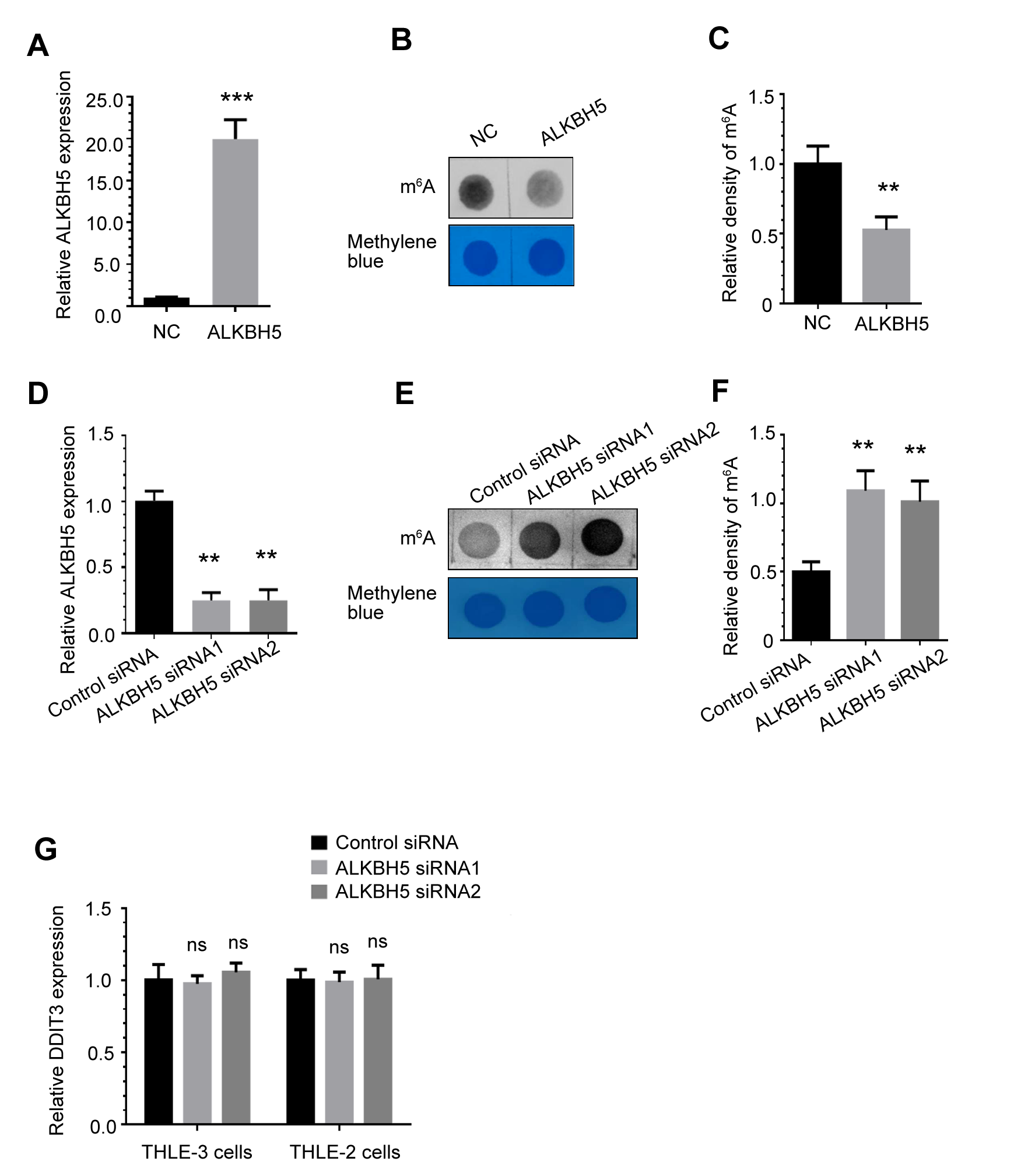


Supplementary Figure 2. **A**,RT-PCR measurements of ALKBH5 expression in THLE-3 cells with overexpressing ALKBH5. **B and C**, The m^6^A methylation level in THLE-3 cells with overexpressing ALKBH5 was detected by m^6^A dot blot assay(**B**) and corresponding quantification(**C**). **D**, Suppressing the targeted gene by ALKBH5 siRNA. **E and F**, The m^6^A methylation level in THLE-3 cells with ALKBH5 knockdown was detected by m^6^A dot blot assay (**E**) and corresponding quantification (**F**). **G**, Effect of ALKBH5 on DDIT3 mRNA expression. Data are means ± SD from three independent experiments. **P* ≤ 0.05; ***P* ≤ 0.01; ****P* ≤ 0.001.


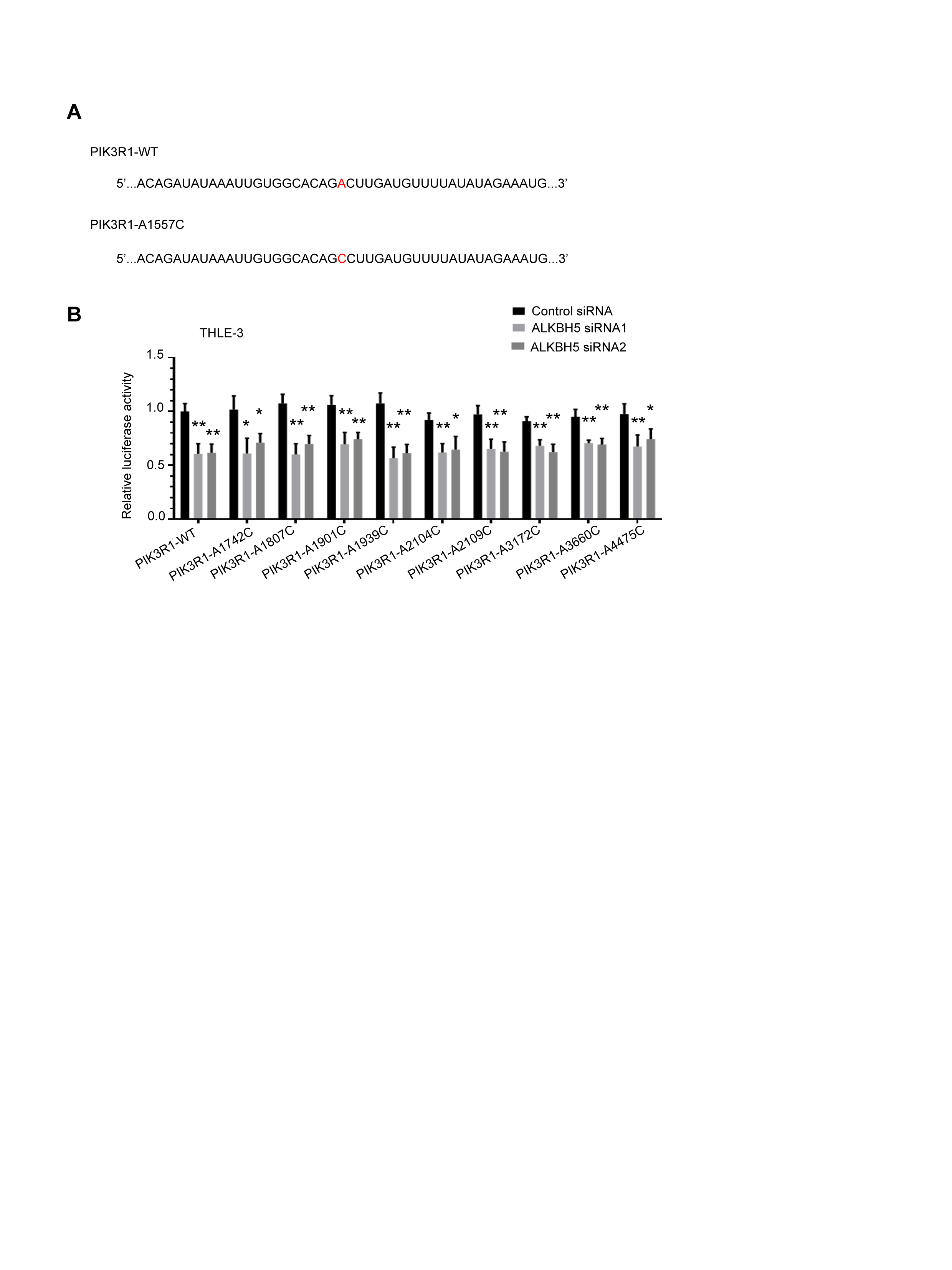


Supplementary Figure 3. **A**, Wild-type or A1557C mutated PIK3R1 sequences. **B**, The effects of m^6^A consensus sequences mutants on the of PIK3R1. Data are means ± SD from three independent experiments. **P* ≤ 0.05; ***P* ≤ 0.01.
