# Supplementary figures and images for: The dual mechanism of m6A demethylase ALKBH5 in regulating energy metabolism during exposure to MC-LR
Source: Cell Death Dis. 2025 Jul 3;16(1):489. doi: 10.1038/s41419-025-07791-x (PMC12229691; doi:10.1038/s41419-025-07791-x)

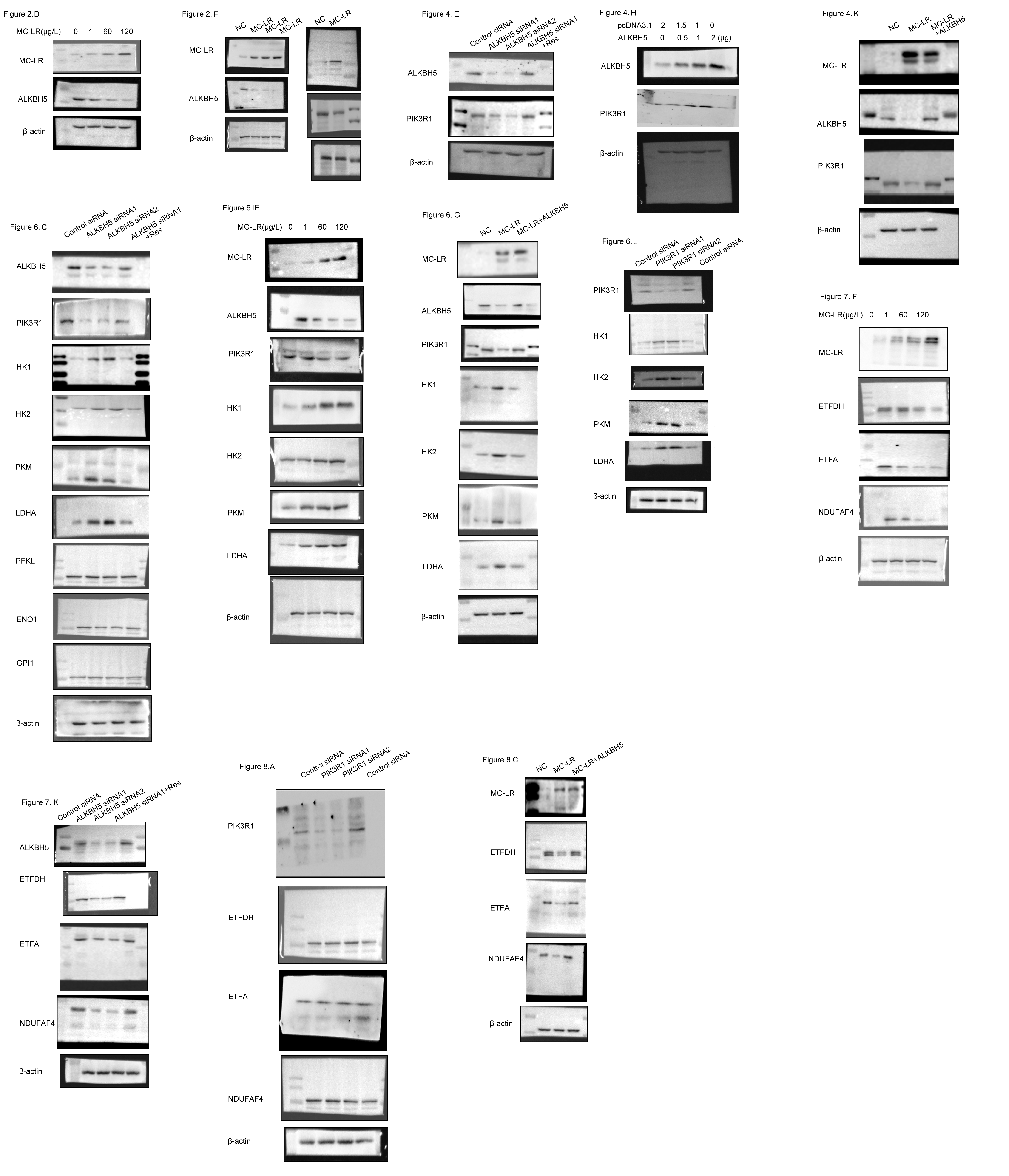

Supplement: Supplementary file 2 — Images of all blots [file 41419_2025_7791_MOESM2_ESM.tif]
